# Risk Factors of Neovascular Glaucoma After 25-gauge Vitrectomy for Proliferative Diabetic Retinopathy with Vitreous Hemorrhage: A Retrospective Multicenter Study

**DOI:** 10.1038/s41598-019-51411-6

**Published:** 2019-10-16

**Authors:** Kei Takayama, Hideaki Someya, Hiroshi Yokoyama, Yoshihiro Takamura, Masakazu Morioka, Seiji Sameshima, Tetsuo Ueda, Shigehiko Kitano, Maki Tashiro, Masahiko Sugimoto, Mineo Kondo, Taiji Sakamoto, Masaru Takeuchi

**Affiliations:** 1 Department of Ophthalmology, National Defense Medical College, Tokorozawa, 3598513 Japan; 2 Department of Ophthalmology, Hyogo College of Medicine, Nishinomiya, 6638501 Japan; 3 Department of Ophthalmology, University of Fukui Faculty of Medical Sciences, Yoshida, 9101193 Japan; 4 Department of Ophthalmology, Kagoshima University, Kagoshima, 8900046 Japan; 5 Department of Ophthalmology, Nara Medical University, Kashihara, 6348522 Japan; 6 Diabetes Center, Tokyo Women’s Medical University School of Medicine, Tokyo, 1628666 Japan; 7 Department of Ophthalmology, Mie University, Tsu, 5148507 Japan

**Keywords:** Outcomes research, Risk factors

## Abstract

Neovascular glaucoma (NVG) is a terminal severe complication in eyes with proliferative diabetic retinopathy (PDR), and PDR eyes with vitreous hemorrhage (VH) which undergo vitrectomy may have higher risk of postoperative NVG. The incidence and the prognostic factor of postoperative NVG after 25-gauge vitrectomy with advanced surgical options remain unclear. We retrospectively reviewed medical records of 268 eyes of 268 consecutive PDR patients with VH who underwent 25-gauge vitrectomy and 12 months follow-up at seven centers. Preoperative ocular factors (visual acuity, tractional retinal detachment, panretinal photocoagulation [PRP]), demographics and clinical factors (sex, age, diabetic duration, HbA1c, hypertension, anticoagulant medication, and kidney function), surgical procedures, and postoperative complications were compared between patients who developed postoperative NVG (9.3%) and those who did not. NVG eyes was significantly younger (*P* = 0.026), had shorter diabetic duration (*P* = 0.022), higher HbA1c (*P* = 0.028), absence of PRP (*P* = 0.039) and higher frequency of postoperative VH (*P* = 0.0075) than non-NVG eyes. Logistic regression analysis identified postoperative VH (*P* = 0.014), shorter diabetic duration (*P* = 0.029), and no PRP (*P* = 0.028) as prognostic factors for postoperative NVG. This multicenter study indicates that younger age, uncontrolled diabetes, no PRP, and postoperative VH are risk factors of post-vitrectomy NVG.

## Introduction

Vitrectomy is conducted to control vitreous hemorrhage (VH) in eyes with proliferative diabetic retinopathy (PDR)^1^. Vitrectomy removes vitreous gels and hemorrhage, and when combined with phacoemulsification and implantation of intraocular lens is also effective for removal of peripheral vitreous gel and hemorrhage^2^. Compared to conventional 20-gauge vitrectomy, 25-gauge microincision vitrectomy system (MIVS) using transconjunctival sutureless cannula decreases surgical invasion, shortens the operating time and duration of hospitalization, and lowers the incidence of intra- and post-operative complications^3^. Postoperative outcomes are further improved by preoperative adjunctive intravitreal anti-vascular endothelial growth factor (VEGF) injection^4^, vitreous visualization using intravitreal triamcinolone acetonide injection^5^, and wide-angle viewing system^6^. These advanced instruments are used worldwide, although postoperative complications may still occur in some cases.

Neovascular glaucoma (NVG) is a terminal severe complication of diabetic retinopathy (DR) and commonly seen in eyes with PDR^7^. Neovascularization is a multi-step process that involves complex interactions of a variety of angiogenic factors, and NVG is caused by growth of a fibrovascular membrane over the trabecular meshwork secondary to a local angiogenic stimulus, which obstructs aqueous outflow^7^. In more advanced stages of neovascularization, connective tissue myofibroblasts associated with neovessel growth contract causing progressive synechial closure of the anterior-chamber angle^7^.

PDR eyes with VH which undergo vitrectomy may have higher risk of postoperative NVG, but the incidence of postoperative NVG after 25-gauge vitrectomy with advanced surgical options remain unclear, and the prognostic factor of postoperative NVG are unknown. The purpose of this study was to evaluate the incidence of postoperative NVG and to identify pre- and intra-operative prognostic factors for postoperative development of NVG in patients with PDR who underwent 25-guage vitrectomy with advanced surgical options.

## Results

### Background of PDR patients with or without postoperative NVG

Twenty-five (9.3%) of 268 patients developed NVG after vitrectomy during 12 months of observation, and the mean period between vitrectomy and developing NVG was 4.2 ± 2.6 months. NVG group (25 patients) comprised 20 males and 5 females, while non-NVG group (243 patients) consisted of 168 males and 75 females. The mean ages of NVG group and non-NVG group were 50.8 ± 12.3 and 55.7 ± 13.4 years, respectively. Table 1 compares the patient baseline characteristics between NVG group and non-NVG group. NVG group was significantly younger (*P* = 0.026), had shorter diabetic duration (*P* = 0.022), higher HbA1c (*P* = 0.028), and preoperative panretinal photocoagulation (PRP) (*P* = 0.039) than non-NVG group. However, there were no significant differences in logMAR, intraocular pressure, or tractional retinal detachment.

**Table 1:** Preoperative demographics, clinical factors, and ocular conditions.

	NVG (n = 25)	non-NVG (n = 243)	P value
Demographics and clinical factors			
Male/female	20/5	168/75	0.26*
Mean age	50.8 ± 12.4	56.1 ± 13.0	0.026^#^
Diabetic duration	9.2 ± 5.9	13.7 ± 10.1	0.022^#^
HbA1c level	8.3 ± 2.3	7.5 ± 1.7	0.028^#^
Estimated glomerular filtration rate	64.2 ± 42.1	62.1 ± 34.2	0.38^#^
Hypertension	16 (64%)	173 (71.2%)	0.45*
Anticoagulation	3 (12%)	39 (16.0%)	0.60*
Ocular conditions			
Baseline logMAR	1.41 ± 0.67	1.44 ± 0.80	0.43^#^
Baseline intraocular pressure	15.3 ± 2.5	14.0 ± 3.3	0.30^#^
Tractional retinal detachment	7 (28%)	92 (37.9%)	0.33*
Panretinal photocoagulation	12 (48%)	171 (70.4%)	0.039

NVG: neovascular glaucoma, HbA1c: glycated hemoglobin. *Analyzed by chi-square test and Fisher’s test, ^#^Analyzed by Mann–Whitney U-test.

### Surgical procedures, postoperative complications in PDR with or without postoperative NVG

Optional surgical procedures performed in combination with 25-gauge vitrectomy in PDR patients with and those without postoperative NVG are shown in Table 2. There were no significant differences in frequencies of preoperative anti-VEGF injection, internal limiting membrane (ILM) peeling, cataract surgery, iatrogenic retinal tear, and tamponade procedure between PDR patients with and without postoperative NVG. As well on subclassified with tamponade materials (air, gas, and silicon oil), there were no significant differences between NVG group and non-NVG group. Postoperative complications in PDR patients with or without postoperative NVG are shown in Table 3. NVG group had a higher rate of postoperative VH than non-NVG (*P* = 0.0075), although there were no significant differences in retinal detachment and epiretinal membrane between two groups.

**Table 2:** Surgical procedures combined with 25-guage vitrectomy in the two groups.

	NVG (n = 25)	non-NVG (n = 243)	P value*
Preoperative anti-VEGF injection	5 (20%)	51 (21.0%)	0.90
ILM peeling	7 (28%)	100 (41.2%)	0.22
Combined cataract surgery	20 (80%)	174 (71.6%)	0.31
Iatrogenic retinal tear	4 (16%)	71 (29.2%)	0.17
Tamponade procedure	9 (36%)	102 (42.0%)	0.55
Air tamponade	4 (20%)	50 (20.6%)	0.59
Gas tamponade	3 (12%)	38 (17.7%)	0.63
Silicon oil tamponade	2 (8%)	14 (5.7%)	0.65

NVG: neovascular glaucoma, ILM: internal limiting membrane. *Analyzed by chi-square test and Fisher’s test.

**Table 3:** Postoperative complications and additional treatment in two groups.

	NVG (n = 25)	non-NVG (n = 243)	P value*
Postoperative complications			
Postoperative VH	12 (48%)	57 (23.5%)	0.0075
Retinal detachment	1 (4%)	11 (4.5%)	0.91
Epiretinal membrane	3 (12%)	11 (4.5%)	0.11

NVG: neovascular glaucoma, VH: vitreous hemorrhage. *Analyzed by chi-square test and Fisher’s test.

### Kaplan-Meier survival analysis and prognostic factors of NVG after vitrectomy for PDR with VH

**Figure 1:**
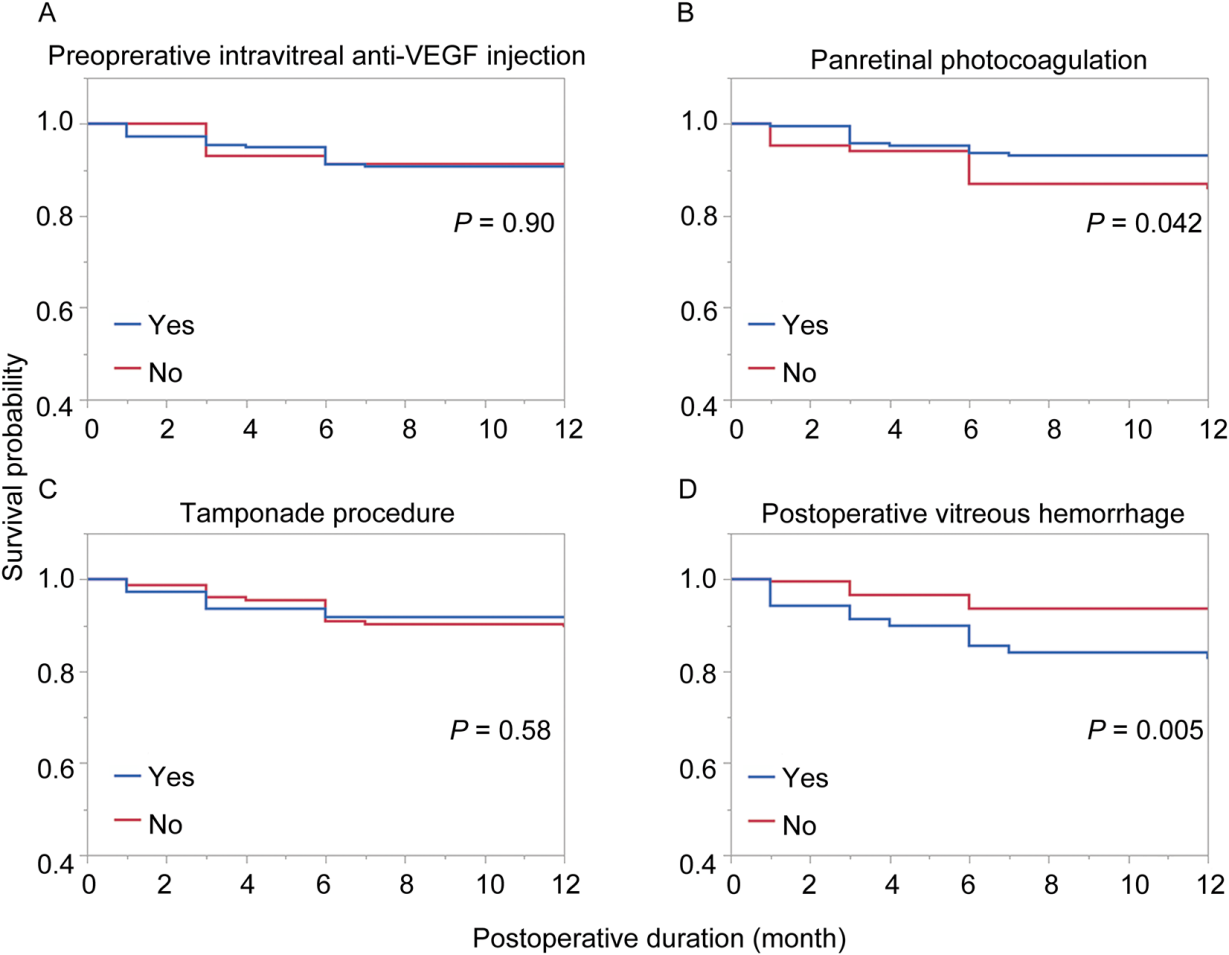
Kaplan-Meier survival rate with or without preoperative intravitreal anti-VEGF injection, panretinal photocoagulation, tamponade procedure, and postoperative vitreous hemorrhage. Generalized Wilcoxon Test detected significant differences in eyes with or without panretinal photocoagulation (**B**) and postoperative vitreous hemorrhage (**D**), not preoperative intravitreal anti-VEGF injection (**A**) or tamponade procedure (**C**).

We evaluated survival rates of NVG with or without preoperative anti-VEGF injection, presence of preoperative PRP, tamponade procedure, or postoperative VH by Kaplan-Meier Methods with generalized Wilcoxon Test and the outcomes were shown in Fig. 1. There were significant differences between eyes with and without preoperative PRP (*P* = 0.042) and postoperative VH (*P* = 0.005), but not in preoperative anti-VEGF injection (*P* = 0.90) or tamponade procedure (*P* = 0.58). Table 4 shows the result of logistic regression analysis to examine the relationship between the development of NVG at 12 months after surgery and age, sex, diabetic duration, HbA1c, preoperative PRP, preoperative intravitreal anti-VEGF, and postoperative VH. The analysis detected a positive correlation of postoperative NVG development with postoperative VH (*P* = 0.014, Odds ration [OR]: 4.51, 95% confidential interval [Cl]: 1.26‒16.2), and a negative correlation with preoperative PRP (*P* = 0.029, OR: 0.22, 95% Cl: 0.05‒0.93) and diabetic mellitus (DM) duration (*P* = 0.029, OR: 0.91, 95% Cl: 0.80–1.00).

**Table 4:** Prognostic factors for neovascular glaucoma after vitrectomy.

Variable	P value	Odds ratio	95% Confidence interval
Age	0.43	1.12	0.77–1.47
Sex	0.30	0.40	0.06–2.26
Diabetic duration	0.029	0.91	0.80–1.00
HbA1c	0.086	1.35	0.97–1.86
Panretinal coagulation	0.028	0.22	0.05–0.93
Anti-VEGF injection	0.78	0.81	0.16–3.85
Postoperative VH	0.014	4.51	1.26–16.2

HbA1c: glycated hemoglobin, VH: vitreous hemorrhage.

## Discussion

The incidence of NVG after vitrectomy in PDR patients with VH enrolled in this study was 9.3%, and was higher than the rates of 6% to 8% reported by previous studies^8–10^. One of the conceivable reasons for the difference is the involvement of VH. Only PDR patients with VH were enrolled in our study, whereas PDR patients without VH were also included in previous reports^11,12^. In fact, Kwon *et al*.^13^ reported that 11.8% of eyes with VH caused by PDR developed postoperative NVG during 12 months after vitrectomy, and their conditions were almost the same as our inclusion criteria. However, the investigators reviewed 127 eyes that underwent 23-gauge vitrectomy by a single retinal specialist in a single center. In contrast, our study was a larger scale (268 eyes) multicenter study using 25-gauge vitrectomy performed by many specialists. Goto *et al*. studied in single center and multi surgeon using 20-gauge and 23-gauge vitrectomy system and mean observation duration was 422 days^9^. Kwon *et al*. studied in single center and single surgeon study with 23-gauge vitrectomy system and observation period was 6 months^13^. The present study is a multicenter study where more surgeons (22 surgeons) participated using all 25-gauge MIVS system with chemical assistant advanced procedures. These results allow us to evaluate risk factors of NVG after vitrectomy with the present procedure for complications of PDR, and to compare with the previous results. The present study may better reflect the current real-world clinical outcome.

In the present study, the NVG group was younger, and had shorter diabetic duration and higher HbA1c, which are compatible with other reports. A previous study demonstrated that younger patients who received vitrectomy for PDR presented more severe anatomical features at the time of vitrectomy and had poorer anatomical success rate with higher rates of postoperative complications including NVG and rapid progression of retinal neovascularization^14^. Similar trends were also found in systemic complications of diabetic patients, such as nephropathy, hypertension, and cardiovascular events^15,16^. Regarding DM duration, the duration of diabetes informed by patients can not present the actual duration. It is well-known that it takes generally more than 10 years to develop DR after the onset of DM. Therefore, 9.2 ± 5.9 years of the mean diabetic duration in NVG group which was shorten than the 13.6 ± 10.1 years in non-NVG group suggested longer duration of uncontrolled DM. From the viewpoint of diabetic control, development of PDR with VH within a shorter diabetic duration indicates poor diabetic control, similar to higher HbA1c levels. It is known that indicators of poor diabetes control are associated with higher risk of diabetic retinopathy^17^, and that control of blood glucose remains the foundation for reducing the risk of retinopathy development and progression^18^.

Logistic regression analyses using development of NVG at 12 months as the dependent variable and including age, sex, diabetic duration, HbA1c, preoperative PRP, preoperative anti-VEGF, and postoperative VH as dependent variables identified absence of preoperative PRP and postoperative VH as prognostic factors for the development of NVG after vitrectomy. Progression of retinal ischemia elicits excessive VEGF production responsible for neovascularization and fibrovascular membrane formation leading to development of PDR^10^. The Diabetic Retinopathy Study^19^ and the Early Treatment Diabetic Retinopathy Study^20^ have established PRP as the primary treatment for proliferative retinopathy. The goal of panretinal photocoagulation, which applies laser burns over the entire retina sparing the central macula, is to promote regression and arrest the progression of retinal neovascularization, possibly by reducing ischemia-driven VEGF production^21^. Previous studies have indicated that VEGF and inflammatory cytokine concentrations are significantly higher in PDR eyes with VH than in those without VH^11,12^. VEGF released from retinal ischemic tissues spread through the aqueous humor to the anterior segment of the eye. The VEGF induces neovascularization of the iris, angle, and connective tissue membrane, followed by synechia of the peripheral iris and trabecular meshwork^22^. Inflammatory cytokines are also elevated in eyes with NVG and PDR^23^, and in addition to growth factors, the inflammatory process is an important cause of iris neovascularization in subjects with NVG^7^. In the present study, postoperative VH was significantly higher proportion in NVG group (12 eyes, 48%) than in non-NVG group (23.5%), in which postoperative VH was observed earlier than development of NVG in all 12 eyes of NVG group. As expected, the neovascularization that remained after vitrectomy should be responsible for postoperative VH, which resulted in NVG. It is conceivable that postoperative VH may increase VEGF and inflammatory cytokines in the aqueous humor resulting in the development of NVG, although no study has compared the concentrations of VEGF and inflammatory cytokines in PDR eyes with or without postoperative VH.

We are aware of limitations of the present study such as the retrospective design, only Japanese participants with type 2 DM, uncertainly duration from developing T2DM to vitrectomy, and difference in skill among surgeons. In surgeon’s skills, although all surgical procedures were performed under similar conditions and data collection was relatively complete, some clinicians could have failed to enter all procedural elements.

In conclusion, this multicenter study demonstrated that the incidence of NVG after 25-gauge MIVS for PDR with VH was 9.3%. Younger patients, absence of preoperative PRP, poor diabetes control, and postoperative VH were prognostic and/or risk factors for the development of NVG after vitrectomy. Comprehensive treatments given jointly by physicians and ophthalmologists are necessary to prevent the development of postoperative NVG and vision loss.

## Material and Methods

A multicenter, retrospective medical chart review study was conducted at seven centers in Japan. This study adhered to the tenets of the Declaration of Helsinki, and was approved by the ethics of committees of the National Defense Medical College Hospital, Hyogo College of Medicine Hospital, University of Fukui Hospital, Kagoshima University Hospital, Nara Medical University Hospital, Tokyo Women’s Medical University School of Medicine Diabetes Center, and Mie University Hospital. Written informed consent was waived by the ethics committees due to the retrospective nature of the study, but all subjects were informed of the study. Medical records at the seven centers were searched to identify PDR patients with VH who underwent 25-gauge vitrectomy and were followed for at least 12 months between March 2010 and December 2016. Eyes with a history of glaucoma including NVG, rubeosis iridis, ocular trauma, past history of vitrectomy, other vitreoretinal disease, uveitis, or preoperative IOP > 21 mmHg were excluded.

Type II DM patients were selected and all patients presented with unresolved VH or VH combined with tractional retinal detachment (TRD) confirmed on B-scan ultrasonography and/or spectral-domain optical coherence tomography (SD-OCT). For PDR patients in which both eyes were operated by unsolved VH, first operated eyes were enrolled in this study. A total of 268 eyes of 268 consecutive PDR patients with VH (188 males and 80 females, mean age 56.8 ± 12.6 years) satisfied the selection criteria. The diagnosis of PDR was determined based on preoperative fundus examination, color photographs, intravenous fluorescein angiograms, SD-OCT, and ultrasonography; or based on intraoperative findings^18^. NVG was defined as the presence of neovascularization in the anterior chamber angle or iris with an IOP higher than 21 mmHg after vitrectomy^7^.

Intravitreal anti-VEGF injection was recommended to the patients as a preoperative optional treatment, although this treatment was excluded in patients with a possibility of retinal detachment shown by B-scan ultrasonography and/or SD-OCT, a history of myocardial infarction or cerebral vascular accidents, or evidence of ocular infection. The surgeon decided which anti-VEGF to use for intravitreal injection.

All patients received peribulbar block under monitored anesthesia care and underwent 25-gauge MIVS with a wide-angle viewing system. Briefly, the surgeon separated the posterior vitreous from the retina by active aspiration using a vitrectomy probe and removed any visible vitreous strands adhering to the retina. Triamcinolone (40 mg/mL; MaQaid; Wakamoto Pharmaceutical, Tokyo, Japan) was injected intravitreally to facilitate visualization and removal of adherent posterior cortical vitreous strands. In all phakia eyes, phacoemulsification and implantation of an artificial intraocular lens using an in-the-bag procedure were performed prior to vitrectomy. The surgeon decided whether to perform ILM peeling using brilliant blue G dye and tamponade procedure with air, gas or silicon oil. No eyes received intravitreal or subtenon triamcinolone acetonide injection, or intravitreal anti-VEGF injection at the end of surgery. Postoperatively, topical antibiotic and anti-inflammatory therapies were administered four times/day for a month. During each visit, the patients underwent a complete ophthalmologic examination including determination of best-corrected visual acuity (BCVA), refractory measurements, intraocular pressure measurements, slit-lamp and dilated fundus observations (with contact and non-contact examination methods), and imaging with SD-OCT. BCVA was measured using a standard Japanese decimal visual acuity chart, and the values were converted to logarithm of the minimum angle of resolution (logMAR) scores for data analysis. The data obtained in the present retrospective study were used in another study conducted by our study group^24,25^.

### Outcomes and statistical analysis

We analyzed preoperative demographic and clinical factors (sex, age, diabetes duration, preoperative HbA1c, status of systemic hypertension, anticoagulation therapy, and estimated glomerular filtration rate), ocular factors (baseline logMAR, baseline intraocular pressure, TRD, and preoperative panretinal photocoagulation), surgical procedures (preoperative intravitreal anti-VEGF injection, ILM peeling, combined cataract surgery, iatrogenic retinal tear, and tamponade procedure), postoperative complications (vitreous hemorrhage, retinal detachment, and epiretinal membrane), and additional postoperative treatment (sub-tenon triamcinolone injection, intravitreal anti-VEGF injection, and repeat vitrectomy).

All results are expressed as mean ± standard deviation for continuous variables. Mann–Whitney U-test, chi-square test and Fisher’s test were used to compare the data between two groups. Kaplan-Meier Methods with generalized Wilcoxon Test was used detect survival probability and significant difference between PDR eyes with or without treatment. for Logistic regression analysis was used to identify prognostic factors for postoperative NVG within 12 months from initial vitrectomy. Estimates of OR and 95% CI were calculated from logistic regression models. All the statistical analyses were performed using JMP Pro ver.14.0 statistical software for Macintosh (SPSS, Chicago, IL).

## References

[1] Berrocal MH, Acaba LA, Acaba A (2016). Surgery for diabetic eye complications. Curr Diab Rep..

[2] Wahab S, Hargun LD (2014). Combined phacoemulsification, vitrectomy and endolaser photocoagulation in patients with diabetic retinopathy and cataract. J Coll Physicians Surg Pak..

[3] Yokota R (2015). Comparison of microinsicion vitrectomy and conventional 20-gauge vitrectomy for severe proliferative diabetic retinopathy. Jpn J Ophthalmol..

[4] Zhang ZH (2013). Vitrectomy with or without preoperative intravitreal bevacizumab for proliferative diabetic retinopathy: a meta-analysis of randomized controlled trials. Am J Ophthalmol..

[5] Enaida H (2003). Possible benefits of triamcinolone-assisted pars plana vitrectomy for retinal diseases. Retina..

[6] Chalam KV, Shah VA (2004). Optics of wide-angle panoramic viewing system–assisted vitreous surgery. Surv Ophthalmol..

[7] Hayreh SS (2007). Neovascular glaucoma. Prog Retin Eye Res..

[8] Sima P, Zoran T (1994). Long-term results of vitreous surgery for proliferative diabetic retinopathy. Doc Ophthalmol..

[9] Goto A (2013). Frequency and risk factors for neovascular glaucoma after vitrectomy in eyes with proliferative diabetic retinopathy. J Glaucoma..

[10] Wakabayashi Y (2012). Intraocular VEGF level as a risk factor for postoperative complications after vitrectomy for proliferative diabetic retinopathy. Invest Ophthalmol Vis Sci..

[11] Nakamura N (2003). Increased concentration of pentosidine, an advanced glycation end product, and interleukin-6 in the vitreous of patients with proliferative diabetic retinopathy. Diabetes Res Clin Pract..

[12] Ehrlich R (2017). Correlation between Interleukin-6 and Thrombin–Antithrombin III Complex Levels in Retinal Diseases. Curr Eye Res..

[13] Kwon J-w, Jee D, La TY (2017). Neovascular glaucoma after vitrectomy in patients with proliferative diabetic retinopathy. Medicine (Baltimore)..

[14] Huang C-H, Hsieh Y-T, Yang C-M (2017). Vitrectomy for complications of proliferative diabetic retinopathy in young adults: clinical features and surgical outcomes. Graefes Arch Clin Exp Ophthalmol..

[15] Pinhas-Hamiel O, Zeitler P (2007). Acute and chronic complications of type 2 diabetes mellitus in children and adolescents. Lancet..

[16] Hillier TA, Pedula KL (2003). Complications in young adults with early-onset type 2 diabetes: losing the relative protection of youth. Diabetes Care..

[17] Fenwick EK (2017). Combined poor diabetes control indicators are associated with higher risks of diabetic retinopathy and macular edema than poor glycemic control alone. PLoS One..

[18] Cheung N, Mitchell P, Wong TY (2010). Diabetic retinopathy. Lancet..

[19] Group ETDRSR (1995). Focal photocoagulation treatment of diabetic macular edema. Relationship of treatment effect to fluorescein angiographic and other retinal charactereistics at baseline. ETDRS report number 19. Arch Ophthalmol..

[20] Group DRSR (1981). Photocoagulation treatment of proliferative diabetic retinopathy: clinical application of Diabetic Retinopathy Study (DRS) findings, DRS Report Number 8. Ophthalmology..

[21] Aiello LP (2005). Angiogenic Pathways in Diabetic Retinopathy. N. N Engl J Med..

[22] SooHoo JR, Seibold LK, Kahook MY (2013). Recent advances in the management of neovascular glaucoma. Semin Ophthalmol..

[23] Kovacs K (2015). Angiogenic and inflammatory vitreous biomarkers associated with increasing levels of retinal ischemia. Invest Ophthalmol Vis Sci..

[24] Someya, Y. *et al*. Internal limiting membrane peeling and 25-gauge for tractional and nontractional diabetic macular edema with proliferative diabetic retinopathy. *J Ophthalmol*. **2019**, 3417425 (2019). 10.1155/2019/5304524 PMC6927058 31885887

[25] Takayama, K. *et al*. Risk factors of revitrectomy for complications in eyes with proliferative diabetic retinopathy: A retrospective multicenter study. (Under submission). 10.1111/aos.14292 31674137

